# Morphometric Characterization of the Domestic Yak Skull (*Bos grunniens* Linnaeus, 1766)

**DOI:** 10.3390/ani16091320

**Published:** 2026-04-25

**Authors:** Hasan Hüseyin Arı, Hasan Alpak, Nariste Kadiraliyeva, Aziz Begaliyev, Milena Djordjevic, Ozan Gündemir

**Affiliations:** 1 Faculty of Veterinary Medicine, Kyrgyz-Turkish Manas University, Bishkek 720044, Kyrgyzstan; hasanh.ari@manas.edu.kg (H.H.A.); nariste.kadyralieva@manas.edu.kg (N.K.); aziz.begaliyev@manas.edu.kg (A.B.); 2 Department of Anatomy, Faculty of Veterinary Medicine, Istanbul University-Cerrahpasa, 34320 Istanbul, Türkiye; alpak@iuc.edu.tr; 3 Department of Anatomy, Faculty of Veterinary Medicine, University of Belgrade, 11000 Belgrade, Serbia; milenadjordjevic@vet.bg.ac.rs

**Keywords:** cranial anatomy, linear measurements, morphological integration, ruminant osteology, sexual dimorphism, skull morphology

## Abstract

This study presents a morphometric characterization of the cranial structure of *Bos grunniens* based on linear measurements taken from adult skulls. Twenty yak skulls (10 males and 10 females) were collected from slaughterhouses in the Bishkek region of Kyrgyzstan. A total of 27 linear cranial measurements were recorded from each specimen, focusing on neurocranial, facial, orbital, and occipital regions. The aim of this study was to describe morphological patterns and structural integration within the yak skull, and to provide baseline morphometric data for a species that remains relatively underrepresented in the literature. Correlation analyses indicated a high degree of morphological association among cranial length, width, and dental parameters. Notably, highly correlated measurement clusters were observed among total cranial length, condylobasal length, dental length, and lateral facial length, indicating coordinated growth and dimensional interdependence in the skull. The first two principal components together explained approximately 75% of the total morphometric variance. PC1 was primarily associated with global skull size and elongation, while PC2 reflected variation in orbital and frontal structures. Additionally, independent *t*-tests revealed statistically significant differences in selected measurements such as total cranial length, dental length, greatest mastoid breadth, greatest inner height of the orbit, and occipital heights, indicating a secondary role of sexual dimorphism in shaping cranial morphology. The findings contribute to anatomical standardization, taxonomic studies, and future comparative morphometric research in large ruminants.

## Simple Summary

Understanding the skull structure of large animals is important for veterinary anatomy and taxonomy. In this study, the skulls of 20 adult yaks from Kyrgyzstan were examined using detailed measurements to better understand how different parts of the skull grow and relate to each other. The results showed that many skull features, such as length, width, and tooth-related measurements, are closely connected and develop in a coordinated way. Two main patterns explained most of the differences between skulls: overall size and shape, and variation in areas around the eyes and forehead. Some differences between males and females were also found, although these were not the main source of variation.

## 1. Introduction

The yak, commonly classified as *Bos grunniens* (domestic yak), while *Bos mutus* refers to the wild form, is a ruminant closely related to cattle (Bos) and bison (Bison), and is adapted to cold, high-altitude environments across China, Tibet, Kyrgyzstan, Mongolia, and neighboring regions [1,2,3]. Yaks play a vital role in local economies and cultures, providing meat, hair, and leather, and serving as pack animals in mountainous areas [4]. As a member of the Bovidae family, the yak includes nearly 20 subspecies, one of which is known as the Kyrgyz yak [5]. These animals are sometimes domesticated in certain regions due to their valuable contributions to human livelihoods.

Composed of several paired bones, the skull forms cavities that enclose and protect the brain and sensory organs essential for perception and balance [6]. Its architecture also houses the initial pathways for the digestive and respiratory tracts. Distinct sutures connect these bones, forming two functional regions: the neurocranial and facial components [6]. The structural organization of the skull illustrates both functional adaptations and taxonomic distinctions within ruminants [7,8]. In these animals, cranial structures like the cornual processes and horn cores demonstrate clear sexual dimorphism, with their size and development closely tied to skull growth and body mass, particularly in early adulthood [9,10]. Consequently, morphometric analyses of the skull offer valuable insights into the functional, developmental, and adaptive dynamics of these structures [11,12]. Such studies enable researchers to quantify variation, assess patterns of integration among cranial regions, and explore how external ecological and selective forces have shaped the yak’s cranial architecture. By mapping these patterns in detail, morphometric studies provide a comprehensive understanding of skull morphology, offering a foundation for comparative anatomy, taxonomy, and even conservation efforts in this unique species [13].

Sexual dimorphism refers to phenotypic differences between males and females of the same species [5]. In many ruminants, these differences manifest in body size and specific cranial features such as horn development [5,9]. For example, domestic cattle are only mildly dimorphic in skull morphology-bulls tend to be larger and heavier with more robust horn bases, whereas cows are smaller with more gracile skulls [9]. Horns are a particularly important sexually dimorphic trait in Bovidae: although both sexes may bear horns, males often have thicker and longer horns, which contribute to broader skull dimensions [5,10]. These cranial differences are not merely ornamental; they reflect adaptations shaped by natural and sexual selection. The head (skull) is a key skeletal structure encoding functional and adaptive information, and head dimorphism can indicate how each sex has adapted to its ecological niche or mating strategies [7,14]. Despite the rich literature on ruminant anatomy, relatively few studies have quantitatively examined sex-based skull differences in these species [15,16,17]. This makes investigations into ruminant skull sexual dimorphism particularly valuable for veterinary anatomy, taxonomy, and adaptive biology. Updated research using modern morphometric techniques (e.g., geometric morphometrics and principal component analysis) has started to uncover subtle shape differences between male and female ruminant skulls that were previously unrecognized [14,15,16,17,18]. In summary, sexual dimorphism in ruminant skulls commonly involves males having larger overall cranial size and more pronounced features (like horn protrusions), although the degree of dimorphism varies widely among species and breeds [19]. Understanding these differences provides insight into each sex’s adaptive roles and is crucial for accurate biological profiling of wild and domestic ruminant populations.

Taxonomic studies play a crucial role in understanding species differentiation, biological diversity, and structural variation among closely related taxa [14,15,16,17]. In this context, cranial morphology is particularly valuable, as the skull provides numerous stable and measurable anatomical landmarks that can be used to distinguish between species or populations. Morphometric analyses of skull structures are widely used in comparative anatomy, systematics, and zooarchaeology, especially when only skeletal remains are available. In addition, variations in cranial morphology may reflect functional adaptations to different environmental conditions and ecological niches. Therefore, detailed cranial measurements contribute not only to anatomical knowledge but also to species identification, classification, and the interpretation of archaeological findings.

While some studies have addressed yak (*Bos grunniens*) phenotypic features, detailed cranial morphometric analyses remain scarce [5], and detailed studies focusing on cranial measurements, particularly in the Tibetan yak, remain scarce. In contrast, advances in shape analysis methods and the widespread application of standardized morphometric measurements have substantially contributed to our understanding of skull morphology in other ruminant species such as cattle, sheep, and water buffalo, as well as bison [14,20,21]. Despite these advancements, there is a notable lack of scientific data regarding the cranial measurements, skull indices, and potential sexual dimorphism in yak populations in Kyrgyzstan. This study was therefore conducted to address this gap by systematically documenting cranial measurements and selected skull indices in Kyrgyz yak specimens, as well as evaluating the extent of intraspecific variation and sexual dimorphism. We hypothesize that, similar to other ruminants, yaks exhibit clear and measurable cranial differences between sexes, which can be identified through comprehensive morphometric analysis. The data generated not only enrich the current scientific literature on yak osteology but also provide a valuable reference for interpreting bone remains from regional excavations and offer a robust basis for future anatomical, adaptive, and archaeological studies.

## 2. Materials and Methods

### 2.1. Samples

**Figure 1:**
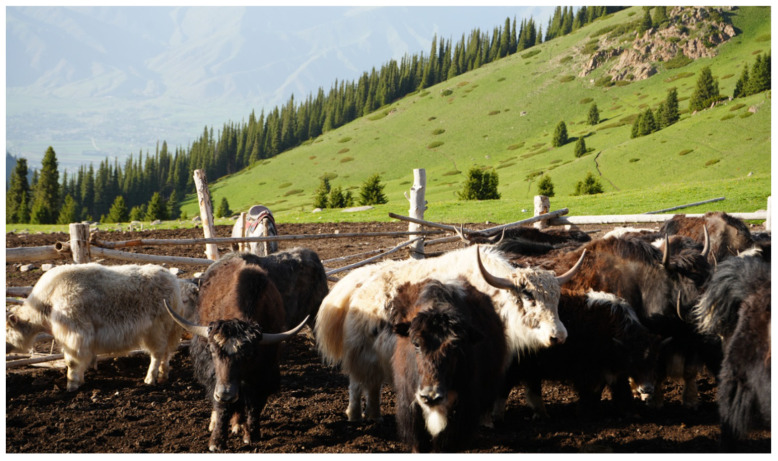
Animals were obtained from slaughterhouses located in Bishkek.

In this study, the material consisted of 20 adult yak (*Bos grunniens*) skulls, including 10 males and 10 females, aged between 3 and 7 years. All animals were considered skeletally mature, thereby minimizing the influence of ontogenetic variation on cranial morphology. The skulls were obtained from slaughterhouses located in Bishkek and its surrounding areas (Figure 1), ensuring a relatively homogeneous environmental background. Ethical approval for the study was granted by the Experimental Animal Ethics Committee of Kyrgyz-Turkish Manas University (Approval Date and Number: 27/11/2023–2023/06).

Following collection, the skulls were transported in sealed containers to the Laboratory of the Department of Anatomy, Faculty of Veterinary Medicine. All procedures were carried out by the research team. Initially, soft tissues such as skin and muscles were removed by dissection. The skulls were then boiled in the faculty’s Boiling Unit to eliminate any remaining soft tissue. Subsequently, residual soft tissues such as ligaments were meticulously cleaned by the researchers in the Anatomy Practice Laboratory. Finally, the skulls were processed in the Polishing Unit using a hydrogen peroxide (H_2_O_2_) and soap solution, then dried and stored under laboratory conditions for the measurements.

### 2.2. Measurement Procedures

**Figure 2:**
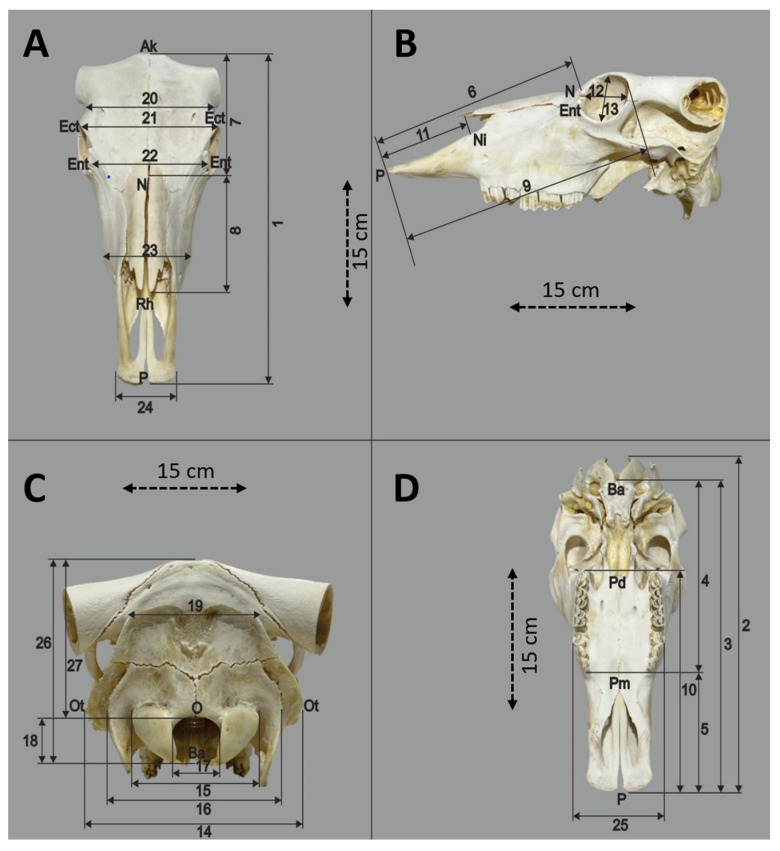
Dorsal (**A**), Lateral (**B**), Caudal (**C**) and Ventral (**D**) view of the skull.

All 20 skulls were measured linearly using a 0.01 mm precision digital caliper. A total of 27 anatomical landmarks and distances were selected based on osteological relevance and morphometric significance. The measurement points, naming conventions, and index calculation formulas were adopted from Ozkan, who conducted a similar morphometric study on water buffalo skulls (Figure 2) [20]. These parameters were chosen to comprehensively reflect cranial length, width, height, and facial dimensions [22].

All measurements were independently performed by two researchers to assess inter-observer reliability. The measurements obtained by both observers were compared, and no statistically significant differences were found between them (*p* > 0.05). This high level of agreement indicates that the measurement protocol is consistent and reproducible. Care was taken to ensure precise placement of the caliper tips at defined osteological landmarks, and skulls with damaged or deformed regions were excluded from the study to maintain data consistency.

The abbreviations and corresponding measurement definitions are listed below:

1. TL—Total cranial length: Distance from the acrocranion to the prosthion.

2. CBL—Condylobasal length: Distance from the occipital condyles to the prosthion.

3. BL—Basal length: Distance from the basion to the prosthion.

4. SSL—Short skull length: Distance from the basion to the premolare.

5. PP—Premolare–prosthion distance.

6. VCL—Viscerocranium length: Distance from the nasion to the prosthion.

7. MFL—Median frontal length: Distance from the acrocranion to the nasion.

8. GLN—Greatest length of the nasals: Distance from the nasion to the rhinion.

9. LFL—Lateral facial length: Distance from the ectorbitale to the prosthion.

10. DL—Dental length: Distance from the postdentale to the prosthion.

11. LLP—Lateral length of the premaxilla: Distance from the nasointermaxillare to the prosthion.

12. GILO—Greatest inner length of the orbit: Distance from the ectorbitale to the entorbitale.

13. GIHO—Greatest inner height of the orbit.

14. GMB—Greatest mastoid breadth: Distance from otion to otion.

15. GBOC—Greatest breadth of the occipital condyles.

16. GBPP—Greatest breadth between the jugular processes.

17. GBFM—Greatest breadth of the foramen magnum.

18. HFM—Height of the foramen magnum: Distance from the basion to the opisthion.

19. LOB—Least occipital breadth: Distance between the most medial points of the temporal grooves.

20. LFB—Least frontal breadth: Distance between the narrowest points of the frontal bone at the orbital borders.

21. GBS—Greatest breadth of the skull: Distance from ectorbitale to ectorbitale.

22. LBO—Least breadth between the orbits: Distance from entorbitale to entorbitale.

23. FB—Facial breadth: Distance between the facial tuberosities.

24. BPOP—Breadth across the premaxillae on the oral protuberances.

25. GPB—Greatest palatal breadth: Distance across the outer alveolar borders of the premolars.

26. GHOR—Greatest height of the occipital region: Distance from the basion to the midpoint of the intercornual ridge.

27. LHOR—Least height of the occipital region: Distance from the opisthion to the midpoint of the intercornual ridge.

In addition to linear measurements, several cranial indices were calculated to express proportional relationships between key anatomical features. These indices were used to express proportional relationships between cranial measurements and to facilitate comparative and ethnological evaluations, as well as to reflect functional adaptations, rather than solely to reduce the effect of overall size. They help highlight relative differences in cranial breadth, height, and facial proportions.

The index formulas were adapted from Ozkan [20] and calculated by dividing one linear measurement by another relevant dimension (e.g., skull length or width), and multiplying the result by 100 to express the value as a percentage. This approach enables meaningful comparisons across individuals by focusing on relative dimensions rather than absolute size alone.

The calculated indices included:

GBS/TL × 100: Greatest breadth of the skull as a percentage of total skull length.

FB/VCL × 100: Facial breadth relative to viscerocranium length.

GBS/VCL × 100: Skull breadth relative to facial length.

GBS/MFL × 100: Skull breadth relative to median frontal length.

GBS/BL × 100: Skull breadth relative to basal length.

GPB/DL × 100: Palatal breadth relative to dental length.

HFM/GBFM × 100: Height of foramen magnum relative to its breadth.

In addition to their mathematical formulation, these indices can be interpreted in relation to commonly used cranial proportions. For example, the ratio of greatest skull breadth to total cranial length (GBS/TL × 100) corresponds to a cranial index, reflecting overall skull shape. Similarly, indices involving facial proportions (e.g., FB/VCL × 100) may be interpreted as facial indices. These indices are useful for comparative evaluation within the same species and may reflect functional adaptations in cranial morphology.

These indices were not included in the multivariate statistical analyses such as PCA and MANOVA, in order to avoid redundancy and preserve the independence of the linear measurements.

### 2.3. Statistical Analysis

All analyses were carried out in R (RStudio 2024.09.1) [23]. To assess potential differences in cranial morphology between male and female yak (*Bos grunniens*), a series of univariate and multivariate analyses were conducted based on 27 linear cranial measurements.

First, descriptive statistics (mean, standard deviation, minimum, and maximum) were calculated for each variable, stratified by sex. Independent samples *t*-tests were then performed to evaluate whether male and female skulls differed significantly in individual measurements. This univariate approach allowed the identification of traits exhibiting statistically significant sexual dimorphism, particularly in dimensions related to cranial length and breadth. Prior to applying the *t*-tests, the normality of distributions and homogeneity of variances were visually inspected and numerically tested where appropriate.

An independent samples *t*-test was used to compare the mean values of cranial measurements between male and female groups. This test evaluates whether there is a statistically significant difference between the means of two independent groups.

Due to the limited sample size, a reduced set of ten cranial measurements was selected for multivariate analysis based on their variability observed in the univariate comparisons. These variables showed the most notable differences between sexes individually. A multivariate analysis of variance (MANOVA) was then applied to assess whether, when considered jointly, these measurements could distinguish male and female skulls in a statistically significant way. MANOVA was chosen as it allows for testing the combined effect of multiple interrelated variables, offering a more holistic view of sexual dimorphism than isolated univariate tests.

To further explore the overall pattern of cranial shape variation and assess whether sexes could be distinguished based on their combined morphometric profiles, a Principal Component Analysis (PCA) was applied to the standardized linear measurements. PCA is a multivariate technique used to reduce dimensionality while preserving as much of the original variance as possible. It was chosen in this study to identify the main axes of shape variation, determine whether males and females formed distinct clusters in morphospace, and evaluate which measurements contributed most strongly to overall variation. Ratio-based indices were excluded from the PCA because they are derived from the original linear measurements, and their inclusion could introduce redundancy and potentially bias the results by over-representing the same morphological information.

In order to examine patterns of association among cranial measurements, Pearson correlation analysis was conducted. This approach allowed the identification of coordinated variation among different skull dimensions. Correlation coefficients were visualized and interpreted to assess structural relationships and potential redundancy among variables. These findings may provide supportive information regarding overall cranial organization, but they do not represent a direct test of morphological integration. Correlation coefficients were interpreted as follows: values of r < 0.30 were considered weak, 0.30–0.59 moderate, 0.60–0.79 strong, and ≥0.80 very strong.

The scores along the first two principal components were used to visualize group separation and assess patterns of variation, while variable loadings on these components were examined to interpret which anatomical features most influenced the observed differences. All statistical significance thresholds were set at α = 0.05.

## 3. Results

### 3.1. Comparative Analysis of Linear Skull Measurements by Sex

In the present study, morphometric comparisons were conducted between male and female skulls of the yak (*Bos grunniens*) based on 27 linear cranial measurements (Table 1, Table 2 and Table 3). The descriptive statistics (mean, minimum, maximum) and independent sample *t*-test results revealed both general trends and statistically significant differences between the sexes.

Male skulls exhibited larger values than females across most cranial length variables, including TL, CBL, BL, and SSL. The TL value showed a statistically significant difference (mean: 458.10 mm in males vs. 420.10 mm in females; *p* = 0.047), while CBL (*p* = 0.075), BL (*p* = 0.087), and SSL (*p* = 0.062) approached significance, indicating a general trend of longer neurocranial dimensions in males. This pattern indicates that males possess a more elongated cranial base, likely reflecting differences in masticatory load.

In the facial region, males also showed larger values in viscerocranium length (VCL), median frontal length (MFL), greatest length of the nasals (GLN), and lateral facial length (LFL), although these were not statistically significant (*p* > 0.05). The dental length (DL), however, was significantly greater in males (mean: 261.20 mm vs. 243.10 mm; *p* = 0.049), suggesting that sexual dimorphism also extends to dental and rostral structures. Additionally, premolare–prosthion (PP) and premaxilla length (LLP) were higher in males, reinforcing this trend.

Males also displayed greater orbital height (GIHO), showing significant sexual dimorphism in orbital dimensions. The greatest inner orbital length (GILO) showed no significant difference (*p* = 0.141), but the consistent direction of size differences supports a pattern of greater orbital capacity in males.

Measurements related to skull breadth showed substantial sexual dimorphism. The greatest mastoid breadth (GMB) was significantly greater in males (175.00 mm vs. 159.10 mm; *p* = 0.0068), indicating a broader neurocranial base. Other transverse parameters such as GBPP, GBS, FB, and LBO were also larger in males, although not all reached statistical significance. The facial breadth (FB) was marginally larger in males (*p* = 0.080), while the greatest breadth of the skull (GBS) approached significance (*p* = 0.094), reflecting a broader cranial and facial structure in males overall.

Sexual dimorphism was particularly evident in the occipital region. Both the greatest height of the occipital region (GHOR) and the least height of the occipital region (LHOR) were significantly greater in males (GHOR: *p* = 0.027; LHOR: *p* = 0.013), reflecting a more vertically developed occipital plane in male skulls. While least occipital breadth (LOB) was also higher in males, this difference was not statistically significant (*p* = 0.139).

No significant sex-based differences were observed in the foramen magnum region. Both the greatest breadth (GBFM) and height (HFM) of the foramen magnum showed nearly identical values between sexes (*p* > 0.94), suggesting that this region may be more functionally constrained and less sexually dimorphic in this species.

Although not statistically significant, male yaks had greater values in breadth across the premaxillae (BPOP) and greatest palatal breadth (GPB), reflecting wider anterior facial structures. These measurements may also be influenced by dental and masticatory loading differences between sexes.

Overall, male skulls exhibited higher values than females in nearly all dimensions, reflecting sexual size dimorphism. Among the measurements, the total length (TL), defined as the distance from acrocranion to prosthion, was significantly longer in males (*p* = 0.047), as were the dental length (DL) (postdentale-prosthion) (*p* = 0.049), and the greatest height of the occipital region (GHOR) (*p* = 0.027) (Table 1). These differences suggest more robust craniofacial development in males, especially in the rostro-caudal and occipital dimensions.

One of the most pronounced differences was observed in the greatest inner height of the orbit (GIHO), the vertical distance between ectorbitale and entorbitale, which was significantly greater in males (*p* = 0.002). This finding may reflect orbital shape dimorphism potentially associated with functional or developmental factors (Table 2).

In terms of proportional indices (e.g., GBS/TL100, FB/VCL100), no significant differences were detected between male and female skulls, suggesting that the observed dimorphism is mainly related to absolute size rather than proportional shape changes (Table 4).

The multivariate analysis of variance (MANOVA) revealed that the combined effect of the selected cranial measurements did not significantly differ between male and female yak skulls (Wilks’ Λ = 0.369, F (10, 9) = 1.54, *p* = 0.264). Although individual measurements showed significant differences in univariate tests, these differences were not sufficient to produce a significant separation in the multivariate analysis. This may be due to the high correlation among cranial variables, which reduces the overall discriminatory power, as well as the relatively small sample size and the limited magnitude of sexual dimorphism across the skull.

This result suggests that, while some traits contribute to sexual dimorphism, the overall multivariate cranial profile across the selected dimensions does not differ significantly between male and female skulls when tested simultaneously.

### 3.2. Principal Component Analysis Results

**Figure 3:**
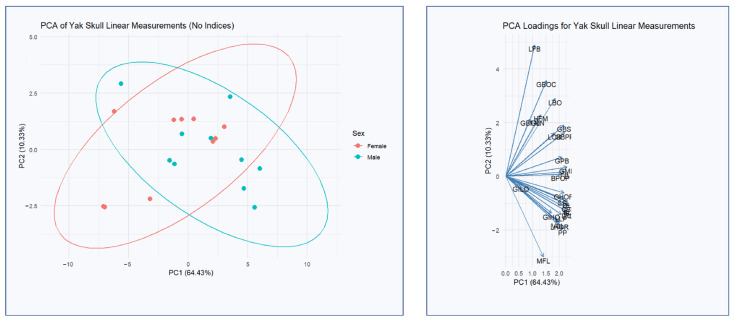
Principal Component Analysis (PCA) of Yak Skull Linear Measurements and Variable Loadings on PC1 and PC2.

Principal Component Analysis based on linear cranial measurements was conducted to explore the main patterns of morphological variation in yak skulls (Figure 3). The first two principal components accounted for a substantial proportion of the total variance, with PC1 explaining 64.43% and PC2 explaining 10.33%, together capturing 74.76% of the total shape variability. The distribution of individuals along PC1 revealed a clear separation between sexes, with male skulls showing a positive mean score (1.74) and female skulls a negative mean score (−1.74). This indicates that PC1 represents the primary axis of sexual dimorphism in cranial morphology. In contrast, PC2 did not differentiate sexes, as both male and female means were near zero, suggesting that this component reflects individual-level variation unrelated to sex.

The variables contributing most strongly to PC1 were those associated with anteroposterior skull elongation and cranial width, such as condylobasal length, basal length, lateral facial length, dental length, greatest mastoid breadth, and total skull length. These findings support the interpretation that males possess generally longer and broader skulls. PC2, on the other hand, was driven more by variables related to cranial height and interorbital breadth, including least frontal breadth, the breadth of the occipital condyles, median frontal length, and the height of the foramen magnum. Although PC2 accounted for a smaller proportion of variance, it still highlights additional aspects of morphological variation likely related to individual or developmental differences rather than sexual dimorphism.

### 3.3. Correlation of Linear Cranial Measurements

**Figure 4:**
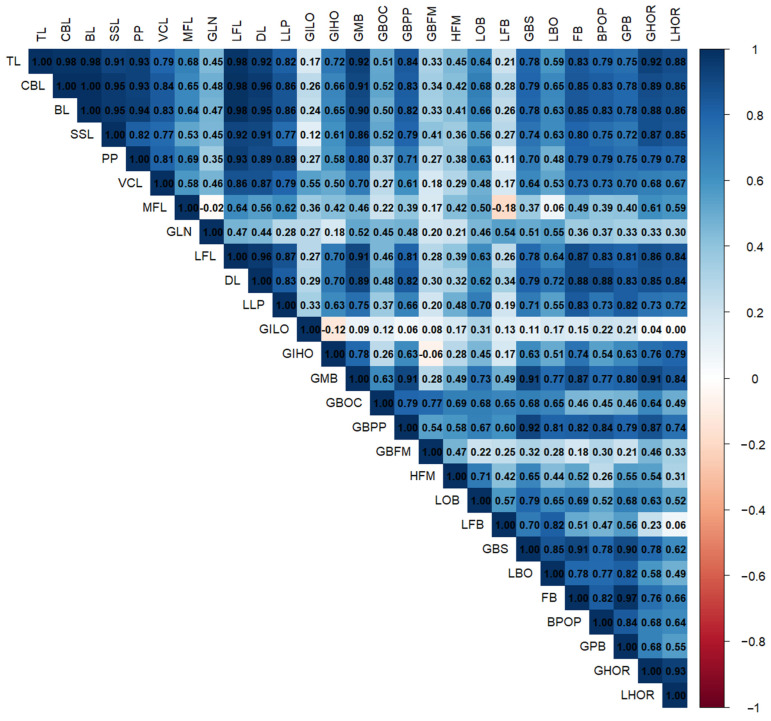
Heatmap showing Pearson correlation coefficients among linear cranial measurements in *Bos grunniens*.

Pearson correlation analysis among the 27 linear cranial measurements revealed several highly significant positive correlations, reflecting coordinated growth patterns among different regions of the skull (Figure 4).

The overall cranial length measurements, total cranial length (TL), condylobasal length (CBL), and basal length (BL) were very strongly correlated with each other (r > 0.98), indicating that these traits likely reflect the same underlying cranial elongation factor. These parameters also strongly correlated with dental length (DL) (r > 0.95), lateral facial length (LFL) (r ≈ 0.98), and greatest mastoid breadth (GMB) (r > 0.90), suggesting that both longitudinal and lateral expansions of the skull are developmentally integrated.

Facial-related measures such as facial breadth (FB), premaxillary width (BPOP), and greatest palatal breadth (GPB) were highly correlated with each other and with DL and LLP (r > 0.85). These patterns suggest that facial width and dental base expansion are closely linked.

A notable integration was observed in the occipital region. The greatest height (GHOR) and least height (LHOR) of the occipital plane were strongly correlated (r = 0.93), and both showed strong associations with TL, BL, and GMB (r > 0.85).

The greatest inner height of the orbit (GIHO) was moderately correlated with many cranial parameters, particularly GMB (r = 0.78) and FB (r = 0.74), suggesting that orbital height expands along with cranial breadth. However, the greatest inner orbital length (GILO) showed low to weak correlations with most variables, and the foramen magnum dimensions (GBFM, HFM) also exhibited relatively low correlations, reflecting functional constraints or lower variability in these regions.

The median frontal length (MFL) displayed only moderate correlations with other variables (r ≈ 0.50 or lower), and greatest nasal length (GLN) showed weak associations with most traits.

## 4. Discussion

The results align with reports from other ruminant studies, where males typically exhibit larger cranial dimensions [14,20,24]. Statistically significant differences were observed in key measurements such as total cranial length (TL), dental length (DL), greatest mastoid breadth (GMB), greatest inner orbital height (GIHO), and occipital height dimensions (GHOR, LHOR). These results confirm that yak skulls exhibit measurable sexual dimorphism, particularly in structures related to cranial elongation and the development of the occipital and mastoid regions. Correlation patterns demonstrated strong interdependence among cranial variables, highlighting coordinated variation across skull regions. The strong positive correlations between these measurements suggest coordinated variation among cranial regions, indicating that these structures develop in a functionally related and proportionally consistent manner. This integration also supports the use of linear cranial measurements as reliable indicators of skull shape variation and sexual dimorphism in yaks. Collectively, these findings contribute new morphometric data for the yak skull, addressing a clear gap in the literature. They provide a valuable reference point for comparative osteological studies of ruminants and offer insights into functional adaptations in yak cranial morphology. Furthermore, this dataset has potential applications in bioarchaeological and taxonomic studies, particularly for the identification of yak remains in Central Asia.

The cranial indices calculated in this study provide additional insight into the proportional relationships within the yak skull. The GBS/TL × 100 ratio, corresponding to a cranial index, indicates that the yak skull exhibits a relatively elongated structure, suggesting a tendency toward a dolichocraniote condition. Similarly, indices involving facial proportions (e.g., FB/VCL × 100) demonstrate balanced relationships between facial width and length, without extreme shortening or widening. However, it should be noted that classifications such as dolichocraniote or brachycranic are primarily defined in human studies, and their direct application to animal species should be interpreted with caution. Therefore, in this study, these indices are used mainly for comparative evaluation and to describe relative cranial shape tendencies rather than strict categorical classification. These findings contribute to a better understanding of cranial proportions in yaks and support their use in comparative anatomical and taxonomic research.

A comparative assessment of the cranial morphometric data highlights notable differences and similarities between yak skulls and those of Simmental and Holstein cattle breeds [25]. The mean total cranial length (TL) was 458.1 mm in male yaks, whereas Simmental and Holstein skulls exhibited larger averages of 485.97 mm and 490.59 mm, respectively. Similarly, basal length (BL) and condylobasal length (CBL) values were higher in cattle, indicating that cattle skulls are generally more elongated compared to yaks. Interestingly, the least occipital breadth (LOB) was markedly smaller in yak skulls (96.42 mm) compared to Simmental (146.6 mm) and Holstein (137.42 mm), underscoring species-specific differences in occipital morphology. These numerical differences may reflect adaptations to environmental pressures, functional demands, or breed-specific selection practices. Although the overall cranial architecture of these ruminants shares common features, the yak’s generally more compact skull dimensions could be linked to its adaptation to harsh alpine conditions and distinct ecological niche.

A comparative analysis of cranial measurements across yaks, water buffalo, and cattle [20] reveals both shared characteristics and notable differences that reflect species-specific adaptations. Cattle skulls (data for cattle based on Ozkan [20] consistently display the largest average dimensions for total length (TL: ~529 mm), condylobasal length (CBL: ~519 mm), and basal length (BL: ~486 mm), indicating generally more elongated cranial structures compared to yaks and water buffalo (TL: ~471 mm). Interestingly, while yaks exhibit shorter skull lengths, they maintain robust mastoid regions (GMB: 175.0 mm), likely linked to adaptations for their cold alpine environments. Water buffaloes, in contrast, have intermediate skull sizes with broad neurocranial dimensions (GMB: 199.23 mm), supporting the development of strong cranial bases for their swamp-grazing habitats. The occipital region height (GHOR and LHOR) also illustrates species-specific differences, with water buffaloes exhibiting more pronounced occipital heights (GHOR: 177.49 mm; LHOR: 168.36 mm) compared to the intermediate values in yaks (GHOR: 143.2 mm; LHOR: 117.4 mm). Yaks also show narrower least occipital breadths (LOB: 96.42 mm for male; 80 mm for female) compared to the wider dimensions observed in cattle (cattle LOB ~136 mm) and water buffaloes (101.38 mm). These differences in cranial morphology likely result from adaptations to environmental and functional demands. The more compact skull of the yak may support survival in alpine conditions, while the broader and more massive skulls of cattle and water buffaloes reflect their different ecological roles and feeding behaviors. Such comparative morphometric studies provide valuable insights into these large ruminants’ functional morphology and offer a strong foundation for future anatomical and archaeological research.

A comparison between the skull morphology of yaks and European bison, informed by the bison study and our linear measurement data on yaks, suggests notable differences in cranial structure that likely reflect species-specific adaptations. It has been reported in previous reference studies that European bison possess broader and more robust skulls compared to domestic cattle [10,21,26]. In our data, yak skulls consistently showed smaller linear measurements compared to cattle, indicating a relatively more compact cranial configuration. Considering that European bison skulls are even broader than cattle skulls, it can be inferred that yak skulls are proportionally smaller and more compact compared to their close relatives, the bison. This more compact skull structure in yaks might be an adaptation to the harsh alpine environments they inhabit, where maneuverability, stability, and thermoregulation are critical for survival. In contrast, the broader skull of the European bison likely reflects the demands of open grassland habitats, where robust cranial structures support social interactions such as combat and display behaviors. Furthermore, while the European bison skull shows pronounced sexual dimorphism in the frontal region to support the growth and use of horns, the yak skull exhibits more modest differences between sexes. This difference might suggest that, in yak social behavior, the role of the horns and the associated cranial structures is less prominent. These findings provide insight into how closely related species within the Bovini group may have developed distinct cranial morphologies to suit their respective ecological niches.

Principal Component Analysis was employed in this study to simplify and interpret the variation present in the 27 linear cranial measurements of yak skulls. This technique is especially valuable when handling multiple variables, as it allows for the identification of the most significant contributors to shape variation. In contrast to the findings of Çakar [25], where total skull length (TL) was identified as the single most influential measurement explaining PC1 variation in cattle skulls, our yak skull analysis revealed a more nuanced pattern. TL did not stand out as the sole dominant measurement in yaks; instead, several measurements-including condylobasal length, basal length, lateral facial length, dental length, and greatest mastoid breadth-contributed similarly to PC1, alongside TL. This suggests that in yaks, cranial shape variation is more evenly distributed across multiple dimensions rather than being primarily driven by overall skull size alone. Such a pattern could indicate species-specific adaptations in cranial morphology in response to environmental and functional demands in high-altitude habitats. Ultimately, these differences may suggest that yak skulls have adapted a more balanced cranial architecture by distributing structural adaptations across multiple regions.

Crucially, shape-focused analyses (e.g., geometric morphometrics combined with PCA) have been instrumental in detecting and visualizing these differences. In Romanov sheep, a 3D geometric morphometric approach identified significant sex-specific shape variation in the cranium, with differences concentrated in regions like the foramen magnum, occipital condyles, and palate [18]. Both manual and AI-assisted landmarking confirmed that rams and ewes have discernible skull shape disparities, although the manual method yielded a clearer separation of sexes in PCA scatterplots [18]. Similarly, in our analysis the PCA revealed that male and female skulls occupy partly distinct regions of morphospace, indicating consistent shape dimorphism. Generally, males tend to have relatively wider skulls (especially across the forehead and zygomatic arches) and longer cranial vaults, while females may have slightly more gracile or short-faced configurations [27]. Such shape differences, albeit subtler than size differences, can be biologically meaningful. They often align with functional demands; for example, a wider male skull can accommodate larger horn bases or stronger jaw musculature.

It is important to note that the magnitude and nature of cranial sexual dimorphism can vary across breeds and it is not always pronounced. For example, in Zell sheep, a morphometric study of 30 skulls (15 males and 15 females) revealed that most cranial measurements did not show statistically significant sex-based differences, except for the distance between the lateral alveolar root and the mental foramen, which was greater in males and allowed an 83.33% accuracy in sex estimation [28]. These findings suggest that in some sheep breeds, sexual dimorphism is limited or highly localized to specific anatomical landmarks. Similarly, a study by Parés Casanova [19] applying geometric morphometric methods to 58 skulls from multiple sheep breeds found only modest discrimination among groups based on cranial shape and size. While shape differences were statistically significant among some regional groups, overall classification success was low (63.8% using shape alone, increasing to 70.6% with size included), indicating that skull morphology offers only limited resolution in breed or sex differentiation among domestic sheep. These results highlight that cranial dimorphism may not be universally strong across ruminants, and its expression likely depends on breed-specific morphological variation and adaptive history.

Females tend to retain more gracile skulls, as their horns, if present, serve less in combat and more in defense or signaling. These sex-specific structural adaptations help explain why males often display greater skull mass, broader zygomatic arches, and thicker cranial walls, consistent with our results. Recent morphometric research also supports this association: in European bison, the frontal bone contributes significantly to sexual differentiation in skull morphology, reflecting its role in supporting horn structures [10,27]. Moreover, in blue wildebeest, overall skull shape correlates with size, and horns exhibit elevated growth rates and variation compared to other skull components, suggesting horns act as a distinct sexually selected module [29]. Additionally, comparative genomic and morphological reviews highlight that horn development is regulated by conserved genetic pathways across Bovidae, providing a mechanistic basis for linked cranial-horn growth [30]. Together, these findings underscore how horn biomechanics, sexual selection, and genetic regulation jointly shape cranial sexual dimorphism in ruminants.

One limitation of the present study lies in the restricted sample size, as only 20 yak skulls could be examined. However, collecting larger numbers of yak skulls poses significant challenges due to the limited availability of specimens, particularly considering the species’ specialized alpine habitats and logistical difficulties associated with field collection. Despite this constraint, the sample size is comparable to those used in previous morphometric studies on large ruminants, where specimen availability is similarly limited. Moreover, the analyses conducted in this study revealed consistent and biologically meaningful patterns of variation, supporting the reliability of the findings. Therefore, the present dataset provides valuable baseline data and an important reference point for future research on yak cranial morphology.

Future studies could expand upon these findings by incorporating geometric morphometric approaches, which would allow for a more detailed analysis of cranial shape variation beyond linear measurements. Additionally, collecting more extensive samples from different age groups would enable investigations into age-related cranial development, which remains largely unexplored in yaks. Such studies would be particularly valuable because they could reveal how yak skull morphology changes with age and how these changes relate to adaptations within their specific living environments. Given the yak’s ecological and cultural importance in these regions, further understanding of cranial growth patterns and functional morphology could have implications for conservation, management, and archaeological studies involving these iconic ruminants.

## 5. Conclusions

This study provides baseline morphometric data on the cranial structure of the Kyrgyz yak (*Bos grunniens*) based on linear measurements and derived indices. The results demonstrate the presence of measurable sexual dimorphism in several cranial dimensions, particularly those related to cranial length and occipital development. The calculated cranial indices indicate that the yak skull exhibits relatively elongated proportions, suggesting a tendency toward a dolichocraniote condition. However, these interpretations are based on proportional relationships and should be considered in a comparative context rather than as strict categorical classifications. Overall, the findings contribute to a better understanding of yak cranial morphology and provide a useful reference for future comparative, anatomical, and taxonomic studies.

## Figures and Tables

**Table 1:** Comparison of Linear Skull Measurements Between Male and Female Yak (*Bos grunniens*).

Measurements (mm)	Mean		Male		Female		*p* Value
	Male	Female	Min	Max	Min	Max	
TL	458.10	420.10	380.0	529.0	379.0	462.0	0.0473
CBL	430.90	402.10	359.0	473.0	358.0	439.0	0.0752
BL	401.60	375.40	336.0	443.0	334.0	409.0	0.0867
SSL	275.40	252.30	223.0	316.0	223.0	280.0	0.0619
PP	131.50	124.50	112.0	147.0	112.0	138.0	0.1226
VCL	259.45	250.20	117.0	302.0	220.0	288.0	0.6315
MFL	175.10	172.70	153.0	201.0	151.0	188.0	0.7197
GLN	192.40	170.10	149.0	300.0	132.0	238.0	0.2015
LFL	336.30	312.60	275.0	372.0	282.0	339.0	0.0605

**Table 2:** Comparison of Linear Skull Measurements Between Male and Female Yak (*Bos grunniens*).

Measurements (mm)	Mean		Male		Female		*p* Value
	Male	Female	Min	Max	Min	Max	
DL	261.20	243.10	211.0	279.0	216.0	267.0	0.0495
LLP	129.10	122.00	102.0	153.0	103.0	153.0	0.2980
GILO	57.50	60.04	49.0	65.6	57.7	63.3	0.1417
GIHO	60.29	53.59	53.8	66.5	48.6	57.0	0.0024
GMB	175.00	159.10	156.0	191.0	142.0	171.0	0.0068
GBOC	94.95	91.50	86.0	100.0	81.0	102.0	0.2468
GBPP	137.20	130.00	128.0	152.0	114.0	142.0	0.0827
GBFM	36.54	36.47	32.5	41.2	31.8	42.0	0.9604
HFM	33.20	33.13	29.0	35.6	28.0	36.5	0.9448

**Table 3:** Comparison of Linear Skull Measurements Between Male and Female Yak (*Bos grunniens*).

Measurements (mm)	Mean		Male		Female		*p* Value
	Male	Female	Min	Max	Min	Max	
LOB	96.42	89.90	80.0	109.2	74.0	99.0	0.1390
LFB	176.00	170.30	168.0	198.0	140.0	187.0	0.3590
GBS	208.35	196.90	192.0	223.0	170.0	221.0	0.0935
LBO	150.10	141.70	137.0	165.0	119.0	163.0	0.1758
FB	136.05	126.40	119.0	147.0	106.0	146.0	0.0804
BPOP	81.46	77.30	66.0	94.0	63.0	92.0	0.3179
GPB	121.60	116.10	109.0	129.0	98.0	135.0	0.2473
GHOR	143.20	132.10	125.0	158.0	121.0	145.0	0.0271
LHOR	117.40	104.60	96.0	131.0	94.0	122.0	0.0128

**Table 4:** Comparison of Cranial Proportional Indices Between Male and Female Yak (*Bos grunniens*).

Indexes	Mean		Male		Female		*p* Value
	Male	Female	Min	Max	Min	Max	
GBS/TL × 100	45.7	46.9	42.1	51.1	42.9	49.6	0.373
FB/VCL × 100	55.4	50.5	46.3	102.0	47.5	52.7	0.367
GBS/VCL × 100	85.5	78.8	70.9	166.0	74.6	84.1	0.482
GBS/MFL × 100	120.0	114.0	104.0	132.0	100.0	127.0	0.249
GBS/BL × 100	52.1	52.5	48.3	57.7	48.4	56.2	0.784
GPB/DL × 100	46.7	47.7	44.7	51.7	44.2	51.0	0.364
HFM/GBFM × 100	91.3	91.1	78.9	102.0	78.6	97.5	0.969

## Data Availability

The datasets generated during and analysed during the current study are available from the corresponding author on reasonable request.
